# Exploration of neural mechanisms underlying antidepressant-like property of *Ziziphora clinopodioides* Lam. essential oil using mouse forced swimming test: Involvement of the monoaminergic systems

**DOI:** 10.1016/j.crphys.2025.100174

**Published:** 2025-11-14

**Authors:** Saeedeh Ghaffarzadeh Shirabad, Samad Alimohammadi

**Affiliations:** Department of Basic Sciences, Faculty of Veterinary Medicine, Razi University, Kermanshah, Iran

**Keywords:** *Ziziphora clinopodioides*, Antidepressant, Forced swimming test, Noradrenergic, Serotonergic, Dopaminergic

## Abstract

**Objective:**

*Ziziphora clinopodioides* has been valued in Iranian traditional medicine for various medicinal applications. This novel study was conducted to assess the antidepressant-like effect of the essential oil of *Ziziphora clinopodioides* (EOZC) and to identify the possible mechanisms contributing to this action through forced swimming test (FST).

**Methods:**

The chemical profile of EOZC obtained by GC-MS. The mice received EOZC (10, 20, and 40 mg/kg) 1 h before the FST intraperitoneally. Moreover, naloxone (non-selective antagonist for opioid receptor subtypes, 1 mg/kg), prazosin (α_1-_adrenergic receptor antagonist, 1 mg/kg), yohimbine (α_2-_adrenergic receptor antagonist, 1 mg/kg), propranolol (β-adrenergic receptor antagonist, 2 mg/kg), WAY100635 (selective 5-HT_1A_ receptor antagonist, 0.1 mg/kg), ondansetron (5-HT_3_ receptor antagonist, 1 mg/kg), haloperidol (non-selective dopamine receptor blocker, 0.2 mg/kg), SCH23390 (selective dopamine D_1_ receptor blocker, 0.05 mg/kg), sulpiride (selective dopamine D_2_ receptor blocker, 50 mg/kg) and flumazenil (GABA_A_/BDZ receptor antagonist, 10 mg/kg) were used to ascertain the neural pathways implicated in the antidepressant-like response of EOZC.

**Results:**

The GC-MS evaluation demonstrated that predominant components of EOZC comprised carvacrol (65.22 %), thymol (19.51 %), p-cymene (4.86 %), γ-terpinene (4.63 %) and E-Caryophyllene (1.07 %). EOZC exhibited a significant dose-dependent effect that resulted in a marked decrease in the duration of immobility time (P < 0.05). The antidepressant-like action of EOZC was reversed by prazosin, yohimbine, WAY100635, ondansetron, haloperidol, SCH23390 and sulpiride. However, this effect remained unaffected by naloxone, propranolol and flumazenil.

**Conclusion:**

These findings indicate that EOZC elicits antidepressant-like response, which relies on its interaction with noradrenergic, serotonergic and dopaminergic pathways.

## Highlights

• The major constituents of *Ziziphora clinopodioides* Lam. essential oil were ascertained.
• *Ziziphora clinopodioides* exhibited a response indicative of antidepressant-like action in the FST.
• The antidepressant mechanism of action of *Ziziphora clinopodioides* essential oil may involve the modulation of the noradrenergic, serotonergic and dopaminergic signaling pathways.

## 1 Introduction

Depression represents a widespread and significant neuropsychiatric condition characterized by profound emotional and physical manifestations. The prevalence of depression constitutes a considerable health challenge for the community, and there exists an immediate necessity to alleviate its burden (Zhuang et al., 2023). Numerous theories have been advanced to elucidate the pathophysiology of depression, encompassing abnormalities in monoamine neurotransmitters function and dysregulation of the hypothalamic-pituitary-adrenal axis. Furthermore, oxidative stress by generating reactive oxygen species (ROS), the secretion of proinflammatory cytokines, or impaired antioxidant defense are believed to contribute to the onset of depression (Teleanu et al., 2022; Correia et al., 2023). The conventional classes of antidepressant drugs exhibit certain constraints regarding their therapeutic efficacy (Papp et al., 2022). Therefore, it is crucial to discern novel antidepressant medications that provide favorable therapeutic responses alongside a reduced incidence of adverse effects. There is a notable increase in interest concerning the application of medicinal plant species in the phytopharmacotherapy of animal models of depressive disorders (Dobrek and Głowacka, 2023).

*Ziziphora clinopodioides,* commonly referred to as Kakouti Kouhi in Persian language, is classified within the Lamiaceae family. This plant exhibits a broad geographical distribution across various regions of Iran, particularly within the western provinces of the country (Mohammadifard and Alimohammadi, 2018). The fresh leaves and stem of *Z. clinopodioides* are employed in traditional Iranian medicine as an appetite stimulant, antiseptic, agent for promoting wound healing, expectorant and sedative (Shahbazi et al., 2016). A recent investigation revealed the antinociceptive activity of *Z. clinopodioides* essential oil in rodent models (Mohammadifard and Alimohammadi, 2018). This plant has been used in therapeutic practices as a pharmacological intervention for hypertension (Senejoux et al., 2012). Moreover, numerous pharmacological investigations have documented the anti-inflammatory (Amini-Shirazi et al., 2009), antimicrobial (Shahbazi and Shavisi, 2016), and antioxidant (Shahbazi, 2017) properties of *Z. clinopodioides*. In addition, the efficacy of *Z. clinopodioides* extract in mitigating Alzheimer's-associated neurological impairments and cognitive deficits induced by ICV administration of STZ in rats has been substantiated (Sedighi et al., 2019). Investigations into the phytochemical composition of *Ziziphora clinopodioides* essential oil have demonstrated its abundance of bioactive compounds, encompassing both monoterpenes and sesquiterpenes (Shahbazi, 2015). Numerous studies have indicated that a subset of these terpenes exhibits a broad range of therapeutic efficacy in the treatment of neuropsychiatric conditions, including but not limited to depression and anxiety, in addition to their role in the regulation of neurotransmitter systems (Zhang et al., 2021; Azimi, 2024). Considering that the antidepressant-like property of *Ziziphora clinopodioides* remain unexamined, the primary goal of current research was to assess the antidepressant-like characteristic of *Ziziphora clinopodioides*, along with the possible mechanistic pathways involved, using the FST in mice.

## 2 Methods

### 2.1 Collection of plant specimen

The newly harvested leaves of *Ziziphora clinopodioides* were collected from the city of Gilan-e-Gharb, located in Kermanshah Province, Iran and subsequently identified by a knowledgeable botanist at the Faculty of Agriculture. A voucher specimen, designated as No. 6816, is preserved in the herbarium of the Research Center of Natural Resources in Tehran, Iran.

### 2.2 Isolation and GC-MS analysis of EOZC

Following collection, the leaves of *Z. clinopodioides* underwent shade drying at ambient temperature (25 ± 2 °C). A total of 100 g of samples were processed by a grinder (Moulinex Co, France) and subsequently combined with 400 ml of distilled water within a glass container. This mixture was then subjected to hydrodistillation for 3.5 h at room temperature, employing a Clevenger-type apparatus. The supernatant was then gathered and dehydrated using 0.5 g of anhydrous sodium sulfate (Merck, Darmstadt, Germany). The essential oil was kept in a dark glass container and wrapped in aluminum foil, maintained at a temperature of 4 ± 1 °C. The chemical constituents of EOZC were ascertained through the application of GC-MS (Thermo Quest Finningan, UK). The GC-MS apparatus comprised 5 % phenyl methyl silicone and 95 % dimethylpolysiloxane, and was outfitted with a DB5 capillary column (30 m, 0.25 mm, film thickness 0.25 μm). A methodology involving electron ionization with an ionization energy set at 70 eV was employed to elucidate the components of the EOZC. Helium, maintained at a steady flow rate of 1.2 mL/min; exhibiting a linear velocity of 29.6 cm/s; with a split ratio of 1:20, was used as the carrier gas. The oven was initially set to a temperature of 50 °C for a duration of 3 min, after which the temperature was increased to 265 °C at a ramp rate of 2.5 °C per min, and ultimately held at 265 °C for 6 min. The injector operated at a temperature of 250 °C. To enhance the precision of the findings, the GC-MS analysis was executed in triplicate.

### 2.3 Chemicals and drugs

The subsequent pharmacological agents were administered: fluoxetine (as a SSRIs), naloxone (non-selective antagonist for opioid receptor subtypes), prazosin (α_1-_adrenergic receptor antagonist), yohimbine (α_2-_adrenergic receptor antagonist), propranolol (β-adrenergic receptor antagonist), WAY100635 (selective 5-HT_1A_ receptor antagonist), ondansetron (5-HT_3_ receptor antagonist), haloperidol (non-selective dopamine receptor blocker), SCH23390 (selective dopamine D_1_ receptor blocker), sulpiride (selective dopamine D_2_ receptor blocker), and flumazenil (GABA_A_/BDZ receptor antagonist) were all obtained from Sigma Chemical Co. (Sigma, Saint Louis, MO). Tween-80 was acquired from Merck Co. (GERBU, Germany). Normal saline served as the medium for the dissolution of all pharmacological agents utilized in the experimental procedures. A range of EOZC doses (10, 20, and 40 mg/kg) was created in a solution of Tween-80 at 0.5 %. The vehicle-treated group was given Tween-80 (0.5 %). All formulations were newly synthesized immediately prior to intraperitoneal (i.p.) delivery at a uniform volume of 10 ml/kg of body weight.

### 2.4 Animals

For this research, adult male albino NMRI mice, aged between 8 and 10 weeks and weighing 25–30 g, were sourced from the Laboratory Animal Facility affiliated with the School of Veterinary Medicine at Razi University. The mice were maintained in a controlled environment with a temperature set at 22 °C ± 2 °C, a relative humidity level of 55 % ± 5 % and a light-dark cycle consisting of 12 h of light and 12 h of darkness. During the course of the experiment, the mice were granted unimpeded access to both nourishment and water. The animals were given a seven-day period for acclimatization prior to the commencement of the research study. It should be emphasized that the Ethics Committee for Animal Welfare at Razi University granted approval for all experimental methodologies executed in this research, with the designated approval number IR.RAZI.REC.1399.029. Furthermore, the aforementioned protocols were implemented in compliance with the established Guidelines for the Care and Use of Laboratory Animals in Scientific Research (Webster, 2014). In order to alleviate the possible influence of circadian fluctuations on behavioral assessments in the FST, the execution of all experimental methodologies took place during the time frame of 08:00 to 12:00 a.m. Every endeavor was undertaken to mitigate the distress experienced by animals and to decrease the quantity of animals utilized in the experimental procedures. Within this study, all animals were applied only once.

### 2.5 Behavioral assessment: forced swimming test (FST)

The progress in antidepressant development relies on the execution of basic behavioral tests conducted with rodents. The FST was extensively utilized for the assessment of rodents behavior in accordance with the previously outlined methodology for mice (Porsolt et al., 1977; Jeyhoonabadi et al., 2007). In this experiment, each mouse was immersed separately in a glass cylinder (height: 25 cm; diameter: 15 cm) that held 10 cm of water kept at a temperature of 25 °C ± 1 °C. Each mouse was permitted to remain within the cylindrical apparatus for a duration of 6 min. Upon the cessation of the mouse's attempts to escape, resulting in a state of floating and stillness within the aqueous environment, the overall length of this immobile phase during the concluding 4 min of the 6 min experimental interval was recorded by a solitary observer. A reduction in the period of immobility serves as a marker for an effect resembling that of antidepressants.

### 2.6 Experimental procedures

**Fig. 1:**
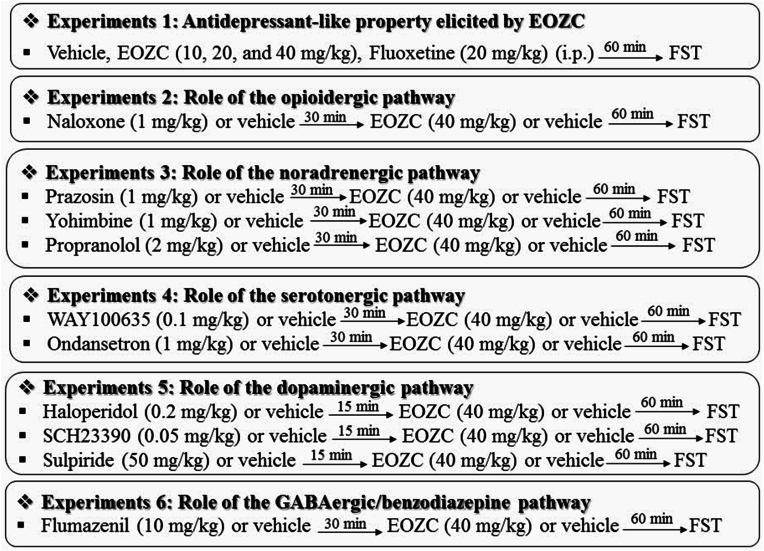
A schematic representation of the experimental design.

A total of six distinct experimental sets were scrutinized in the FST assay in the following manner. A schematic representation of the experimental design is shown in Fig. 1. In experiment 1, to evaluate the potential antidepressant-like properties, the mice were administered with vehicle, EOZC (10, 20, and 40 mg/kg) or fluoxetine (20 mg/kg) 60 min prior to the FST. Consequently, the most effective concentration of EOZC was determined for the subsequent experimental protocols. To explore the mechanisms by which EOZC induces an antidepressant-like effect, experiments 2–6 were conducted. In experiment 2, an investigation was carried out to examine the possible role of the opioidergic system in the antidepressant-like activity of the EOZC. To accomplish this objective, mice were administered naloxone (1 mg/kg) or vehicle as a pretreatment. Following a 30 min interval, they were given EOZC (40 mg/kg) or vehicle, and subsequently underwent the FST 60 min later. In experiment 3, to evaluate the interaction of the EOZC with the noradrenergic system, mice were pretreated with prazosin (1 mg/kg), yohimbine (1 mg/kg) and propranolol (2 mg/kg) or vehicle and after 30 min they were administered EOZC (40 mg/kg) or vehicle and subsequently assessed in the FST 60 min later. The objective of experiment 4 was to examine the possible contribution of the serotonergic system to the antidepressant-like action elicited by EOZC. To this end, mice were pretreated with WAY100635 (0.1 mg/kg) and ondansetron (1 mg/kg) or vehicle and 30 min later received EOZC (40 mg/kg) or vehicle followed by the FST after 60 min. In experiment 5, we further examined the impact of the dopaminergic system on the antidepressant-like property of EOZC. For this purpose, animals were pretreated with haloperidol (0.2 mg/kg), SCH23390 (0.05 mg/kg) and sulpiride (50 mg/kg) or vehicle and after 15 min they received EOZC (40 mg/kg) or vehicle. 60 min later, the FST was performed. The purpose of experiment 6 was to examine the potential role of the GABAergic/benzodiazepine system in the antidepressant-like effect induced by EOZC. To achieve this objective, mice were administered flumazenil (10 mg/kg) or vehicle; 30 min subsequent to this pretreatment, they were given EOZC (40 mg/kg) or vehicle, followed by the FST after a 60 min interval. The dosages of all drugs utilized in the present study, as well as the schedules for their administration, were established in accordance with prior reports and the authors' unpublished pilot investigations (Mohammadifard and Alimohammadi, 2018; Gu et al., 2012; Colla et al., 2012, 2014; Savegnago et al., 2007; Pałucha-Poniewiera and Pilc, 2012).

### 2.7 Statistical analysis

The gathered data were expressed as the mean ± standard error of mean (SEM). The dataset underwent examination through either one-way or two-way analysis of variance (ANOVA), accompanied by Tukey's HSD post-hoc examination, employing the SPSS software version 21 for Windows (SPSS, Inc. Chicago, IL, USA). The criterion for statistical significance was defined as P < 0.05.

## 3 Results

**Fig. 2:**
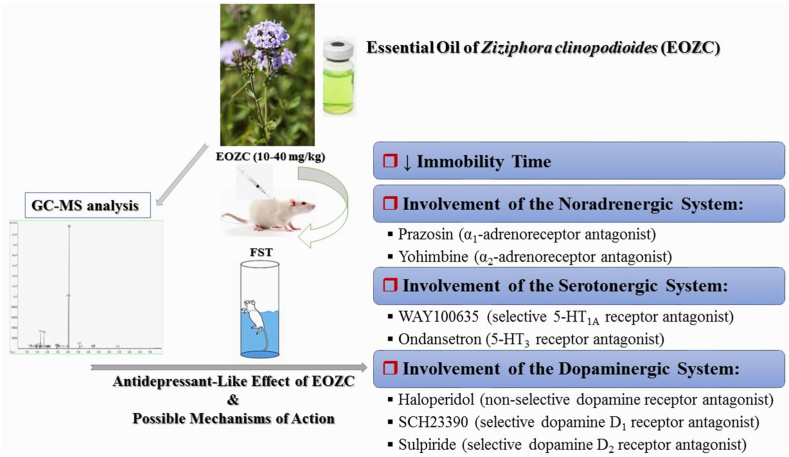
A comprehensive summary of the findings from the current research.

Fig. 2 presents a comprehensive summary of the findings derived from the current research endeavor. The specifics of these findings are elaborated upon in the subsequent sections.

### 3.1 GC-MS analysis of the EOZC

**Fig. 3:**
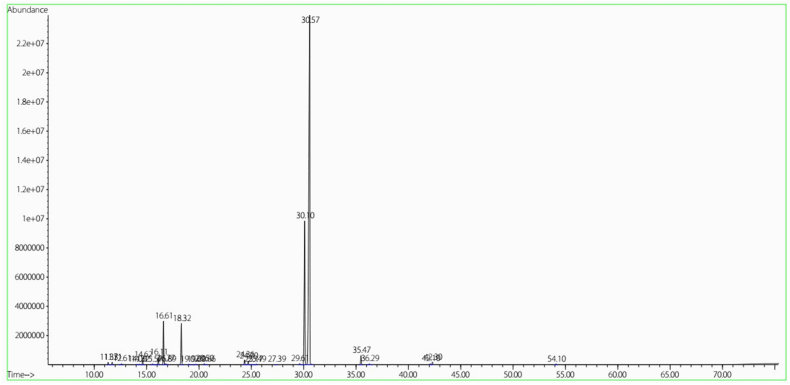
GC-MS chromatogram of the essential oil of *Ziziphora Clinopodioides* (EOZC).

The properties of the constituents found in the EOZC are delineated in Table 1. The chromatogram is illustrated in Fig. 3. Based on the findings from the GC-MS analysis, EOZC comprised 24 distinct compounds, accounting for 99.65 % of its total composition. With respect to the chemical constituents, the primary elements of the EOZC were identified as carvacrol (65.22 %), thymol (19.51 %), p-cymene (4.86 %), γ-terpinene (4.63 %) and E-Caryophyllene (1.07 %) (Table 1). A total of 95.29 % of the yield can be attributed to these five components.

**Table 1:** The chemical composition of the essential oil of *Ziziphora Clinopodioides* (EOZC).

N	Compound	Composition (%)	Retention Time (min)	Kovats Index
1	Carvacrol	65.22	30.57	1315
2	Thymol	19.51	29.61	1293
3	p-cymene	4.86	16.61	1030
4	γ-terpinene	4.63	18.32	1063
5	E-Caryophyllene	1.07	35.47	1427
6	α-Terpinene	0.79	16.11	1021
7	Borneol	0.61	24.36	1183
8	Myrcene	0.51	14.62	992
9	Terpinene-4-ol	0.48	24.7	1190
10	Caryophyllene oxide	0.31	42.30	1595
11	α-Pinene	0.27	11.71	934
12	α-Thujene	0.26	11.33	927
13	Linalool	0.13	20.5	1105
14	Camphene	0.13	12.61	952
15	α-Phellandrene	0.13	15.58	1010
16	Spathulenol	0.12	42.10	1590
17	β-Phellandrene	0.11	16.89	1036
18	Limonene	0.1	16.77	1033
19	α-Terpineol	0.08	25.49	1206
20	Terpinolene	0.08	19.69	1089
21	1-Octen-3-ol	0.08	14.32	986
22	cis-Sabinene hydrate	0.07	19.02	1077
23	β-Pinene	0.06	14.06	981
24	Carvacrol, methyl ether	0.04	27.38	1246
	Total	99.65

### 3.2 Antidepressant-like property elicited by EOZC on mice assessed in the FST

**Fig. 4:**
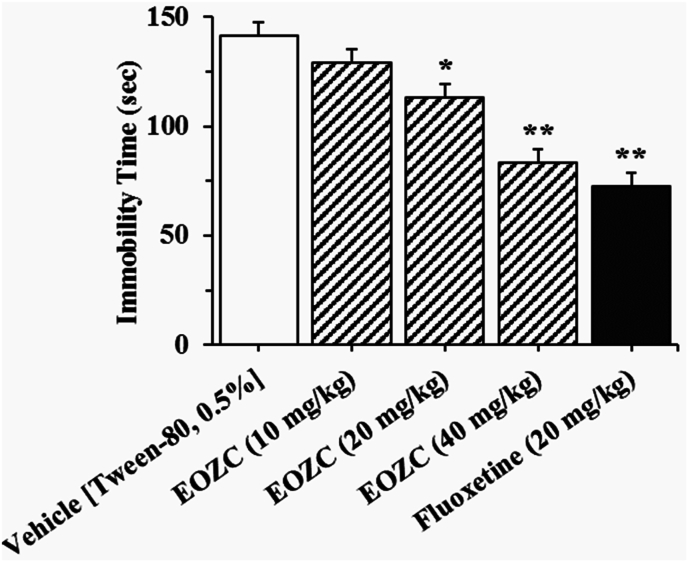
The effect of the i.p. administration of essential oil of *Ziziphora clinopodioides* (EOZC) (10, 20 and 40 mg/kg) and fluoxetine (20 mg/kg) on the immobility time in the forced swimming test (FST) in male mice. Data are expressed as mean ± SEM (n = 8). Data were analyzed using one-way ANOVA followed by Tukey's HSD post-hoc multiple comparison test. ∗P < 0.05 and ∗∗P < 0.01 when compared to vehicle treated group.

As depicted in Fig. 4, EOZC (20 and 40 mg/kg) resulted in a dose-dependent reduction in the duration of immobility observed during the FST, alongside fluoxetine (20 mg/kg), when compared to the group receiving the vehicle treatment. One-way ANOVA demonstrated a notable influence of the EOZC in the FST [F(4,35) = 21.97, P < 0.01]. When the 10 mg/kg dose of EOZC was evaluated, no difference was detected (P > 0.05).

### 3.3 Exploration of various potential mechanisms underlying the EOZC-induced antidepressant-like effect in the FST

Given that the administration of EOZC (40 mg/kg) demonstrated the most significant level of efficacy, all further investigations aimed at elucidating the mechanisms underlying the antidepressant-like effects of the EOZC were performed utilizing this specific dosage.

#### 3.3.1 Role of the opioidergic pathway in the antidepressant-like effect induced by EOZC

**Fig. 5:**
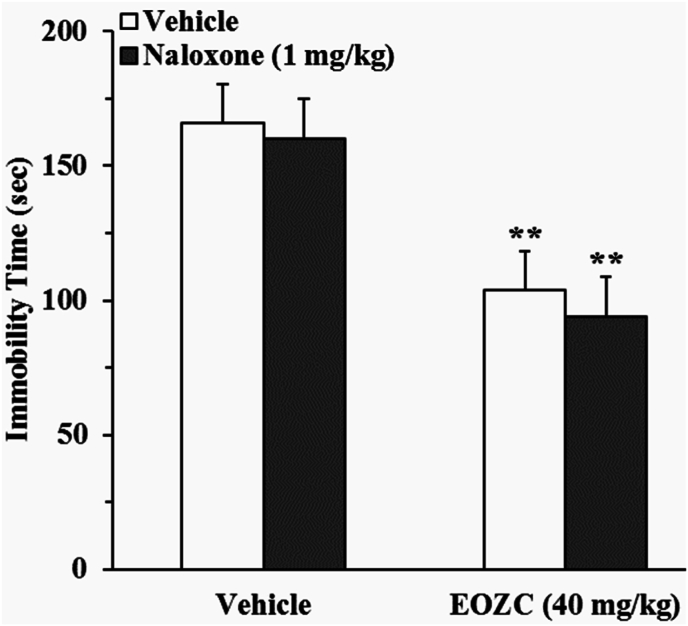
Effect of the pretreatment of mice with naloxone (1 mg/kg, a non-selective opioid receptors antagonist, i.p.) on antidepressant-like effect elicited by EOZC (40 mg/kg, i.p.) in the FST. Data are expressed as mean ± SEM (n = 8). Statistical analysis was performed by two-way ANOVA followed by the Tukey's HSD post-hoc multiple comparison test. ∗∗P < 0.01 when compared to vehicle-treated group.

As shown in Fig. 5, pretreatment of mice with naloxone did not succeed in inhibiting the anti-immobility effect observed with EOZC. A two-way ANOVA indicated a statistically significant effect of EOZC treatment [F(1,28) = 71.24, P < 0.01], whereas naloxone pretreatment did not demonstrate a significant effect [F(1,28) = 1, P > 0.05], nor did the interaction between naloxone pretreatment and EOZC treatment [F(1,28) = 0.07, P > 0.05].

#### 3.3.2 Role of the noradrenergic pathway in the antidepressant-like impact produced by EOZC

**Fig. 6:**
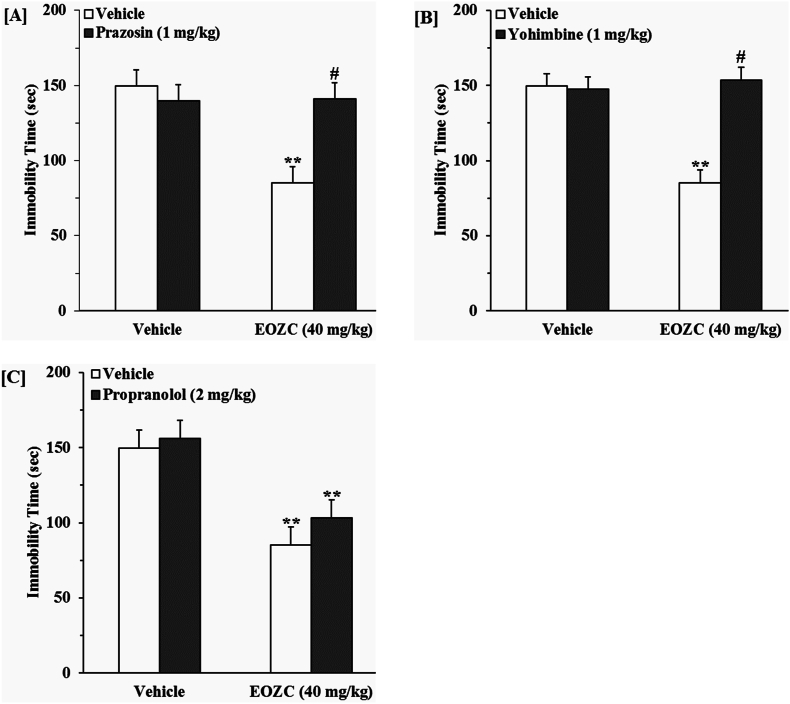
Effect of the pretreatment of mice with prazosin (1 mg/kg, an α_1_-adrenoreceptor antagonist, i.p., panel A), yohimbine (1 mg/kg, an α_2_-adrenoreceptor antagonist, i.p., panel B) and propranolol (2 mg/kg, a β-adrenoreceptor antagonist, i.p., panel C) on antidepressant-like effect induced by EOZC (40 mg/kg, i.p.) in the FST. Data are expressed as mean ± SEM (n = 8). Statistical analysis was performed by two-way ANOVA followed by the Tukey's HSD post-hoc multiple comparison test. ∗∗P < 0.01 when compared to vehicle-treated group; #P < 0.01 as compared to the EOZC group pretreated with vehicle.

The findings illustrated in Fig. 6A indicate that the pretreatment of animal with prazosin inhibited the antidepressant-like action caused by the EOZC. A two-way ANOVA revealed significant differences of prazosin pretreatment [F(1,28) = 8.83, P < 0.01], EOZC treatment [F(1,28) = 16.75, P < 0.01] and prazosin pretreatment × EOZC treatment interaction [F(1,28) = 18.16, P < 0.01]. Fig. 6B illustrates that the pretreatment of mice with yohimbine effectively inhibited the antidepressant-like efficacy of EOZC. A two-way ANOVA revealed significant differences of yohimbine pretreatment [F(1,28) = 21.51, P < 0.01], EOZC treatment [F(1,28) = 16.50, P < 0.01] and yohimbine pretreatment × EOZC treatment interaction [F(1,28) = 24.36, P < 0.01]. Fig. 6C illustrates that the administration of propranolol prior to the experiment did not inhibit the antidepressant-like impact of EOZC. A two-way ANOVA indicated significant difference of EOZC treatment [F(1,28) = 70.58, P < 0.01], but not of propranolol pretreatment [F(1,28) = 3.11, P > 0.05] and propranolol pretreatment × EOZC treatment interaction [F(1,28) = 0.68, P > 0.05].

#### 3.3.3 Role of the serotonergic pathway in the antidepressant-like action induced by EOZC

**Fig. 7:**
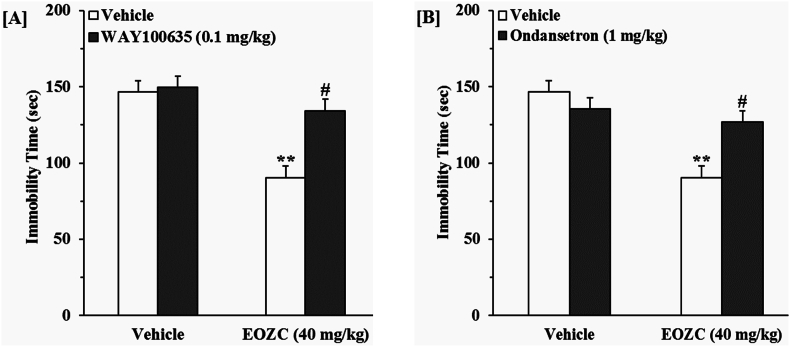
Effect of the pretreatment of mice with WAY100635 (0.1 mg/kg, a selective 5-HT_1A_ receptor antagonist, i.p., panel A) and ondansetron (1 mg/kg, a 5-HT_3_ receptor antagonist, i.p., panel B) on antidepressant-like effect induced by EOZC (40 mg/kg, i.p.) in the FST. Data are expressed as mean ± SEM (n = 8). Statistical analysis was performed by two-way ANOVA followed by the Tukey's HSD post-hoc multiple comparison test. ∗∗P < 0.01 when compared to vehicle-treated group; #P < 0.01 as compared to the EOZC group pretreated with vehicle.

The findings illustrated in Fig. 7A indicate that the pretreatment of mice with WAY100635 suppressed the antidepressant-like response elicited by the EOZC. A two-way ANOVA revealed significant differences of WAY100635 pretreatment [F(1,28) = 9.76, P < 0.01], EOZC treatment [F(1,28) = 22.73, P < 0.01] and WAY100635 pretreatment × EOZC treatment interaction [F(1,28) = 7.51, P < 0.05]. Fig. 7B demonstrates that the pretreatment of animal with ondansetron diminished the antidepressant-like efficacy of EOZC. A two-way ANOVA revealed notable differences associated with ondansetron pretreatment [F(1,28) = 5.82, P < 0.05], EOZC treatment [F(1,28) = 19, P < 0.01] and ondansetron pretreatment × EOZC treatment interaction [F(1,28) = 10.08, P < 0.01].

#### 3.3.4 The contribution of the dopaminergic pathway to the antidepressant-like effect induced by EOZC

**Fig. 8:**
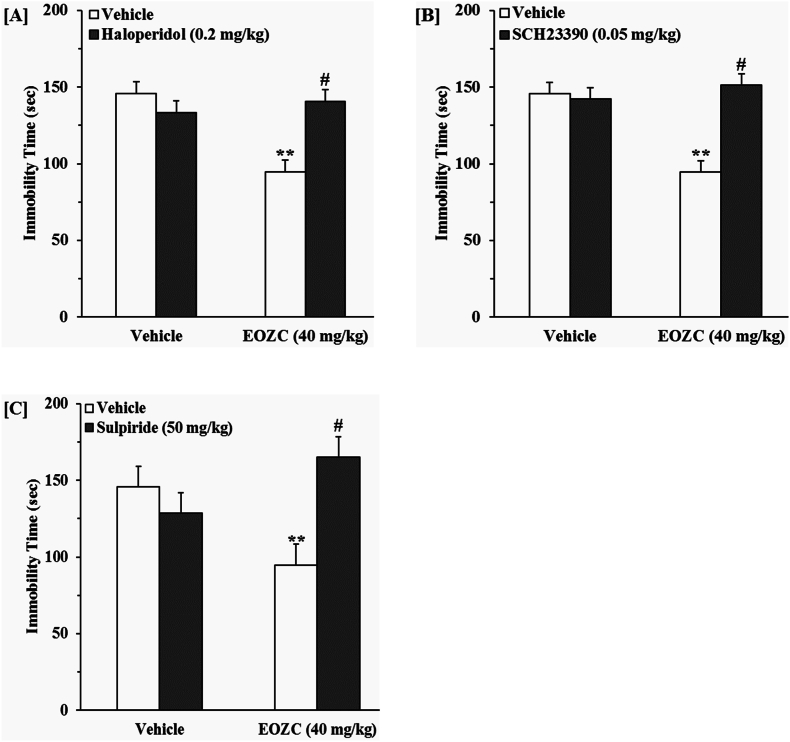
Effect of the pretreatment of mice with haloperidol (0.2 mg/kg, a non-selective dopamine receptor antagonist, i.p., panel A), SCH23390 (0.05 mg/kg, a selective dopamine D_1_ receptor antagonist, i.p., panel B) and sulpiride (50 mg/kg, a selective dopamine D_2_ receptor antagonist, i.p., panel C) on antidepressant-like effect induced by EOZC (40 mg/kg, i.p.) in the FST. Data are expressed as mean ± SEM (n = 8). Statistical analysis was performed by two-way ANOVA followed by the Tukey's HSD post-hoc multiple comparison test. ∗∗P < 0.01 when compared to vehicle-treated group; #P < 0.01 as compared to the EOZC group pretreated with vehicle.

As shown in Fig. 8A, the pretreatment of mice with haloperidol was able to block the antidepressant-like response elicited by the EOZC. A two-way ANOVA revealed significant differences of haloperidol pretreatment [F(1,28) = 4.61, P < 0.05], EOZC treatment [F(1,28) = 7.85, P < 0.01] and haloperidol pretreatment × EOZC treatment interaction [F(1,28) = 13.93, P < 0.01]. Fig. 8B shows that the administration of SCH23390 prior to the experiment blocked the anti-immobility activity of EOZC. A two-way ANOVA revealed significant differences of SCH23390 pretreatment [F(1,28) = 13.37, P < 0.01], EOZC treatment [F(1,28) = 8.36, P < 0.01] and SCH23390 pretreatment × EOZC treatment interaction [F(1,28) = 16.75, P < 0.01]. The data presented in Fig. 8C indicates that the administration of sulpiride prior to the experiment was capable of negating the anti-immobility impact linked to EOZC. A two-way ANOVA indicated notable variances attributable to sulpiride pretreatment [F(1,28) = 12.73, P < 0.01], EOZC treatment [F(1,28) = 10.95, P < 0.01], and the interaction between sulpiride pretreatment and EOZC treatment [F(1,28) = 34.47, P < 0.01].

#### 3.3.5 The function of the GABAergic/benzodiazepine system in the antidepressant-like influence elicited by EOZC

**Fig. 9:**
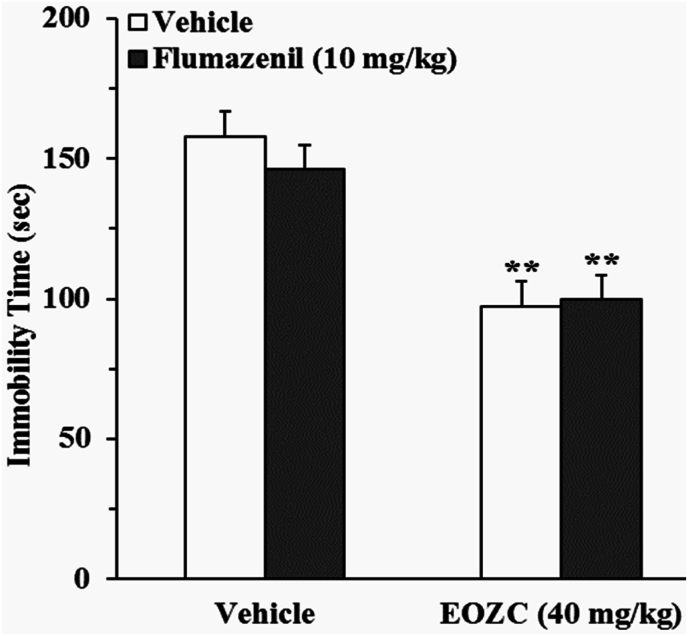
Effect of the pretreatment of mice with flumazenil (10 mg/kg, a GABA_A_/BDZ receptor antagonist, i.p.) on antidepressant-like effect elicited by EOZC (40 mg/kg, i.p.) in the FST. Data are expressed as mean ± SEM (n = 8). Statistical analysis was performed by two-way ANOVA followed by the Tukey's HSD post-hoc multiple comparison test. ∗∗P < 0.01 when compared to vehicle-treated group.

As shown in Fig. 9, pretreatment of mice with flumazenil did not influence antidepressant-like activity of EOZC. A two-way ANOVA revealed a statistically significant effect of EOZC treatment [F(1,28) = 48.37, P < 0.01], whereas flumazenil pretreatment did not show a significant effect [F(1,28) = 0.37, P > 0.05], nor did the interaction between flumazenil pretreatment and EOZC treatment [F(1,28) = 0.88, P > 0.05].

## 4 Discussion

Depression is classified as a disorder distinguished by an array of emotional, cognitive, and somatic manifestations. The prevalence of depression constitutes a significant public health concern that is on the rise (Zhong et al., 2024). Traditional herbal medicine has demonstrated efficacy in addressing a diverse array of health issues, including depressive disorders, and is gaining wider acceptance on a global scale (Dai et al., 2022). To the fullest extent of the authors' comprehension, this research provides the first report concerning the exploration of possible mechanisms that may elucidate the antidepressant-like characteristics of the EOZC, employing the FST in mice. The results distinctly demonstrated that the intraperitoneal administration of the EOZC led to a considerable decline in duration of immobility in a dose-dependent fashion. This suggests that EOZC may serve as a promising antidepressant candidate for the management of depression. Based on the findings obtained through GC-MS analysis in this research, the primary constituents of the EOZC were recognized as phenolic compounds comprising carvacrol (65.22 %), thymol (19.51 %), p-cymene (4.86 %), γ-terpinene (4.63 %) and E-Caryophyllene (1.07 %). Oxidative stress and dysregulation of the HPA axis have been identified as significant contributors to neuropsychiatric conditions, such as depression. Furthermore, the presence of neuroinflammation and neurotransmitter disturbances are correlated with the manifestation of depressive disorders (Correia et al., 2023).

Phenolic compounds, recognized as bioactive molecules, exhibit both antioxidant and antidepressant properties. For instance, following carvacrol administration in experiments using mice, an effect resembling that of antidepressant medications was noted (Melo et al., 2011; Valentim et al., 2024). Thymol acts as an antioxidant (Monteiro et al., 2007) and alleviates depressive symptoms in CUMS mice by enhancing neurotransmitter levels and suppressing proinflammatory cytokine expression (Deng et al., 2015). p-cymene monoterpene has shown anti-inflammatory effect by inhibiting NF-κB and MAPK signaling pathways (Zhong et al., 2013), and its antioxidant properties contribute to its neuroprotective effects in the mouse brain (de Oliveira et al., 2015). γ-Terpinene is associated with reduced inflammation and alleviated neuropathic pain by lowering proinflammatory cytokines and pain responses (Ramalho et al., 2015; Bilbrey et al., 2022). On the other hand, E-Caryophyllene which is another component of the EOZC, has been noted for its ability to reduce inflammation in mice (Bento et al., 2011) as well as in vitro antioxidant activity (Al-Qudah, 2016). Ethnopharmacological research has further established that β-Pinene and Linalool exhibit antidepressant-like activity in mice behavioral model of depression (Guzmán-Gutiérrez et al., 2015). In light of the previously discussed information, it can be inferred that the presence and accessibility of phenolic compounds within EOZC may plausibly play a role, to some degree, in the antidepressant-like effect identified in the present study.

A further objective of the present research was to clarify the possible mechanisms that contribute to the antidepressant-like property of EOZC with particular emphasis on the engagement of opioidergic, noradrenergic, serotonergic, dopaminergic, and GABAergic/benzodiazepine pathways. The participation of the opioidergic system in the pathology of depressive disorders and the mechanisms of action of antidepressants in rodent models has been thoroughly elucidated in prior investigations, alongside its significant function in nociceptive processes (Brüning et al., 2011). Nevertheless, the results obtained from the current study revealed that the pretreatment with naloxone in mice did not negate the anti-immobility effect of EOZC. This suggests that the antidepressant-like action induced by EOZC is not facilitated through interaction with the opioid system.

The monoaminergic hypothesis concerning depression is a broadly acknowledged theoretical framework (Elhwuegi, 2004). It is widely recognized that monoamine neurotransmitters, including noradrenaline, serotonin, and dopamine within the CNS, serve a fundamental role in the etiology of depressive disorders. The ideal therapeutic efficacy of antidepressants is associated with the enhancement of monoaminergic network functionality, which constitutes the primary objective in the innovation of novel pharmacological agents (Millan, 2004). A substantial body of clinical and experimental evidence indicate a connection between the central noradrenergic system and occurrence of depression (Itoi and Sugimoto, 2010). There exists considerable evidence supporting the contribution of α_1_ and α_2_-adrenoreceptors in the pharmacological mechanisms underlying the efficacy of both pharmaceutical agents and botanical substances exhibiting antidepressant property (Pytka et al., 2016; Olubodun-Obadun et al., 2023). For instance, the inhibitory influence on mobility exerted by desipramine was mitigated by the administration of prazosin (Danysz et al., 1986). In a separate investigation, yohimbine could block the antidepressant-like activity of clonidine (O'Neill et al., 2001). In the current study, pretreatment of mice with prazosin and yohimbine successfully prevented the antidepressant-like effect caused by the EOZC in FST, indicative that EOZC exerts its antidepressant-like action through interaction with α_1_ and α_2_ adrenergic receptors. Nonetheless, the lack of a mitigative effect on the antidepressant-like property induced by EOZC following pretreatment with propranolol suggests that the engagement of EOZC with β-adrenoceptors is unlikely to account for its antidepressant-like property.

A plethora of research has confirmed the engagement of the serotonergic system in the etiology of depression, highlighting 5-HT and its receptors as primary focal points for therapeutic intervention (Lin et al., 2023). Numerous antidepressant agents are presently accessible, ostensibly functioning through the serotonergic pathway. The 5-HT_1A_ receptors are found at both presynaptic and postsynaptic locations, and there is evidence indicating their essential role in mediating antidepressant-like responses in behavioral assessments (O'Neill and Conway, 2001). A deficiency in the operational capacity and expression of the 5-HT_1A_ receptors has been documented in individuals experiencing depression (Kaufman et al., 2016). The 5-HT_3_ receptors are recognized as a promising target in the context of depression. Nevertheless, the involvement of 5-HT_3_ receptors in depressive disorders has been significantly less investigated compared to that of 5-HT_1A_ (Brüning et al., 2011). The 5-HT_3_ receptor agonists appear to mitigate the efficacy of antidepressants in non-clinical models, in contrast, 5-HT_3_ receptor antagonists, exemplified by ondansetron, exhibit activities reminiscent of antidepressants (Bétry et al., 2011). In our research, the anti-immobility action triggered by EOZC was effectively abolished when mice were pretreated with both WAY100635 and ondansetron. These data imply that the serotonergic system, particularly through the 5-HT_1A_ and 5-HT_3_ receptors, is likely involved in the antidepressant-like response of EOZC.

It is widely recognized that the dopaminergic system is closely linked to depressive disorders and its related symptoms (D'Onofrio et al., 2024). According to the most recent studies, dopamine and its receptors distribution in different brain regions may represent a significant therapeutic objective for alleviating symptoms of depression (Zhao et al., 2022). In this regard, activation of dopamine D_1_ receptor enhances neurogenesis within the hippocampus and demonstrates an antidepressant-like effect in rats (Mishra et al., 2019). Furthermore, the administration of a dopamine D_1_ receptor antagonist abolished the antidepressant-like effect of chlorpheniramine observed in the mouse TST (Hirano et al., 2007). Additionally, the antidepressant-like action of pramipexole observed in rats is facilitated through the engagement of D_2_ and D_3_ receptor subtypes (Breuer et al., 2009). Likewise, bromocriptine, recognized as a prominent D_2_ receptor agonist, demonstrated efficacy in alleviating depressive manifestations in a patient exhibiting treatment-resistant depression (Wada et al., 2001). Considering the influence of dopaminergic pathway in anti-depressant responses, another finding uncovered during our investigation demonstrated that the pretreatment of animals with haloperidol, SCH23390 and sulpiride resulted in the prevention of EOZC-induced antidepressant-like action.

Apart from the dysregulation of monoaminergic systems in depressive disorders, GABAergic/benzodiazepine system plays a crucial role in the modulation of behaviors associated with depression. Postmortem investigations have revealed that depressive disorders are distinguished by alterations in GABAergic/benzodiazepine neurotransmission (Kalueff and Nutt, 2007; Ghose et al., 2011). Nevertheless, the outcomes derived from the current investigation provide empirical data that robustly indicate the EOZC-induced antidepressant-like action was not antagonized in the presence of flumazenil. This implies that the antidepressant-like activity of the EOZC do not occur via the GABAergic/benzodiazepine pathway.

## 5 Conclusion

The results of this study demonstrate that the essential oil of *Ziziphora clinopodioides* (EOZC) induces an antidepressant-like effect and this outcome is dependent on the dosage administered. Moreover, in light of these observations, it can be deduced that the aforementioned antidepressant-like effect is, to some extent, modulated by the participation of noradrenergic (α_1_ and α_2_ adrenoreceptors), serotonergic (5-HT_1A_ and 5-HT_3_ receptors), and dopaminergic (D_1_ and D_2_ receptors) pathways. However, further research is required to precisely identify the molecular targets and pathways in the CNS affected by EOZC, especially in comparison to conventional antidepressants. Additionally, it is important to evaluate the efficacy, safety, and pharmacokinetics of EOZC in long-term and clinical settings to better understand its therapeutic potential and applicability.

## Author contributions

Saeedeh Ghaffarzadeh Shirabad: Conceptualization, Data curation, Formal analysis, Investigation, Methodology. Samad Alimohammadi: Conceptualization, Formal analysis, Investigation, Methodology, Project administration, Supervision, Validation, Writing – original draft, Writing – review and editing. All authors approved the final version of the manuscript.

## Funding

This research was supported by a grant from the Research Council of the Faculty of Veterinary Medicine, Razi University, Iran.

## Declaration of competing interest

The authors declare that they have no conflicts of interest to disclose.

## Data Availability

The data that support the results of this study are available from the corresponding author upon reasonable request.
